# Re-learning mental representation of walking after a brain lesion. Effects of a cognitive-motor training with a robotic orthosis

**DOI:** 10.3389/fnbot.2023.1177201

**Published:** 2023-07-31

**Authors:** Maria-Chiara Villa, Giuliano C. Geminiani, Marina Zettin, Alessandro Cicerale, Irene Ronga, Sergio Duca, Katiuscia Sacco

**Affiliations:** ^1^BraIn Plasticity and Behavior Changes (BIP) at Department of Psychology and Neuroscience Institute of Turin (NIT), University of Turin, Turin, Italy; ^2^Clinical Psychology Unit, Molinette Hospital, Città della Salute e della Scienza, Turin, Italy; ^3^Centro Puzzle-Rehabilitation of Acquired Brain Damages, Turin, Italy; ^4^Department of Environment, Land and Infrastructure Engineering (DIATI), Polytechnic of Turin, Turin, Italy; ^5^Neuroradiology Unit, Koelliker Hospital, Turin, Italy

**Keywords:** robotic orthosis, cognitive training, functional magnetic resonance imaging (fMRI), walking, rehabilitation, stroke

## Abstract

**Introduction:**

Stroke-related deficits often include motor impairments and gait dysfunction, leading to a limitation of social activities and consequently affecting the quality of life of stroke survivors. Neurorehabilitation takes advantage of the contribution of different techniques in order to achieve more benefits for patients. Robotic devices help to improve the outcomes of physical rehabilitation. Moreover, motor imagery seems to play a role in neurological rehabilitation since it leads to the activation of the same brain areas as actual movements. This study investigates the use of a combined physical and cognitive protocol for gait rehabilitation in stroke patients.

**Methods:**

Specifically, we tested the efficacy of a 5-week training program using a robotic orthosis (P.I.G.R.O.) in conjunction with motor imagery training. Twelve chronic stroke patients participated in the study. We evaluated balance and gait performance before and after the training. Six of them underwent fMRI examination before and after the training to assess the effects of the protocol on brain plasticity mechanisms in motor and imagery tasks.

**Results:**

Our results show that the rehabilitation protocol can effectively improve gait performance and balance and reduce the risk of falls in stroke patients. Furthermore, the fMRI results suggest that rehabilitation is associated with cerebral plastic changes in motor networks.

**Discussion:**

The present findings, if confirmed by future research, have the potential to advance the development of new, more effective rehabilitation approaches for stroke patients, improving their quality of life and reducing the burden of stroke-related disability.

## 1. Introduction

Stroke is a primary cause of disability worldwide, with approximately 15 million people experiencing a stroke each year (Feigin et al., 2017). One of the most debilitating consequences of a stroke is difficulty on walking, or gait dysfunction. Stroke occurs suddenly and may result in major deficits (Stokes, 2004), thus often drastically hampering patients' quality of lives, by leading to a loss of autonomy, and consequently, of social relationships. Stroke survivors are therefore often affected by a series of complex problems on a physical, psychological, and social level. One of the most common and greater needs of a stroke patient is to restore walking ability, since motor deficits often persist after discharge from the hospital (Pennycott et al., 2012) and greatly limit independence in carrying out the activities of daily and social life (Hesse, 2008). The main difficulties in walking for stroke patients are decreased strength, inability to produce voluntary muscle contractions, and inappropriate muscle activity (Perry et al., 1995). Furthermore, a few weeks following brain injury, two additional deficits may occur, such as spasticity and changes in the mechanical properties of the muscles that cause abnormal stretching of the muscle groups (Olney and Richards, 1996). Stroke patients may also present somatosensory and proprioceptive dysfunctions, that prevent them from receiving an adequate sensory feedback from their movements, thus impairing the normal gait cycle (Kessner et al., 2019).

Motor rehabilitation in the neurological field must take into consideration all these different aspects and must be implemented as a multidisciplinary approach to improve impaired functions, reduce symptoms, and increase the personal wellbeing of both the patient and their family, according to the World Health Organization guidelines (WHO). Recovery is a complex process that occurs through a combination of spontaneous and learning-dependent processes. These include restoration of the functionality of the damaged tissue, reorganization of the neural pathways to relearn the lost function, and compensatory mechanisms that help patients interact with the environment through new skills (Langhorne et al., 2011). Neuro-motor rehabilitation techniques are then based on the assumption that the brain can recover or compensate for lost functions through the phenomenon of cerebral plasticity. It has been demonstrated that, while acquiring new skills, cortical regions associated with sensorimotor functions of the body parts most involved in the task gradually start to be represented over larger cortical territories (Pascual-Leone et al., 1994; Kami et al., 1995). Thus, it is likely that, after a brain injury, the sensorimotor experiences of the individual can remodel the structure and function of undamaged parts of the brain, promoting the recovery process (de Diego et al., 2013; Chen and Shaw, 2014). One possibility to support this process is to passively induce lower limb movements. Such passive leg movement has been demonstrated to induce a proprioceptive and kinesthetic activation which provides an afferent input to the supra-spinal motor control centers, stimulating and re-activating these circuits (Rossini et al., 2003; Li et al., 2018).

In recent years, more and more robotic devices have been developed to help the patient regain confidence in gait movements in complete safety (Hobbs and Artemiadis, 2020). These devices can be used in combination with a Body Weight Support (BWS) system, allowing weight relief during patients' movement (Bruni et al., 2018). Several studies have been made to experiment the effectiveness of these devices and some of these seem to give positive results, in association with classical therapy (Hesse, 2008; Mehrholz et al., 2020).

Another factor that seems to play a crucial role in improving motor functions in both healthy subjects and patients during post-stroke rehabilitation is Motor Imagery (from now on, MI) (Sacco et al., 2006; Zimmermann-Schlatter et al., 2008; Li et al., 2018). MI is defined as “an active process during which the representation of an action is internally reproduced within working memory without any overt output” (Decety and Grèzes, 1999). MI seems to be effective for motor rehabilitation both from a behavioral perspective (Jackson et al., 2001), but also for promoting cerebral reorganization in motor rehabilitation of stroke patients (Sun et al., 2013).

Here, we implemented a protocol that combines the use of MI with a robotic exoskeleton, in the context of gait rehabilitation. The robotic orthosis used in this rehabilitation protocol is called Pneumatic Interactive Gait Rehabilitation Orthosis (P.I.G.R.O.) (Belforte et al., 2014; Sacco et al., 2018), it is specifically developed for mimicking natural walking movements, and it can be used in both passive and active modes in order to assist patients during the motor rehabilitation process. The objective of this study is thus to verify whether a combined MI+PIGRO protocol is effective in rehabilitating the gait of chronic stroke patients and whether it can actively support brain plasticity. With this aim, we conducted a 5-week training program combining the use of a robotic orthosis and MI training for gait rehabilitation in chronic stroke patients. We tested behavioral performance before and after the rehabilitation protocol. Following the training, we expected an improvement in gait and balance, assessed by behavioral scales. Furthermore, we expected that our combined rehabilitation protocol may promote brain plasticity. To verify this, before and after the rehabilitation training, we examined areas of cerebral activation while performing foot movement and MI task during fMRI scanning.

## 2. Materials and methods

### 2.1. Participants

Twelve patients with outcomes of chronic ischemic and hemorrhagic stroke (M = 7; age range = 33–75), were recruited at least one year after the cerebral event. Six of them had a right hemisphere lesion, while the other six had a left hemisphere lesion (for a broad description of lesions see Table 1). They were preliminary tested by a cognitive screening to determine if exclusion criteria were present. They we excluded in case of cognitive deterioration, aphasia, psychiatric illness or severe behavioral changes, drug or alcohol abuse, severe deficits in one or more of the following areas: visual, auditory, attentional, reasoning, language comprehension, presence of neglect, apraxia. Physical inclusion criteria were hemiparesis in the lower limbs, unstable gait but preservation of the ability to stand independently or with assistance. Physical exclusion criteria were the presence of peripheral neuropathies of the lower limbs, spinal lesions, previous central nervous system disease, and severe orthopedic impairment. Seven subjects were recruited from the ASL of Fossano (CN) and five were recruited from the Puzzle center in Turin.

**Table 1:** Description of subjects' lesion.

**Subject**	**Hemisphere**	**Type of lesion**	**Location**
Sub-01	Left	Mixed	Frontal
			Temporal
Sub-02	Left	Mixed	Frontal
			Temporal
			Parietal
Sub-03	Left	Cortical	Frontal
Sub-04	Left	Mixed	Frontal
			Temporal
			Parietal
Sub-05	Left	Cortical	Frontal
			Parietal
Sub-06	Left	Cortical	Temporal

Mixed lesion refers to a lesion involving both cortical and subcortical areas, while cortical to lesion involving just cortical areas.

The research has been conducted in accordance with the World Medical Association's Declaration of Helsinki. It has been performed in accordance with national guidelines and with the prior approval of the Ethics committee of “AOU Città della Salute e della Scienza di Torino”. The patients provided their written informed consent to participate in this study.

### 2.2. The robotic gait orthosis—P.I.G.R.O.

**Figure 1:**
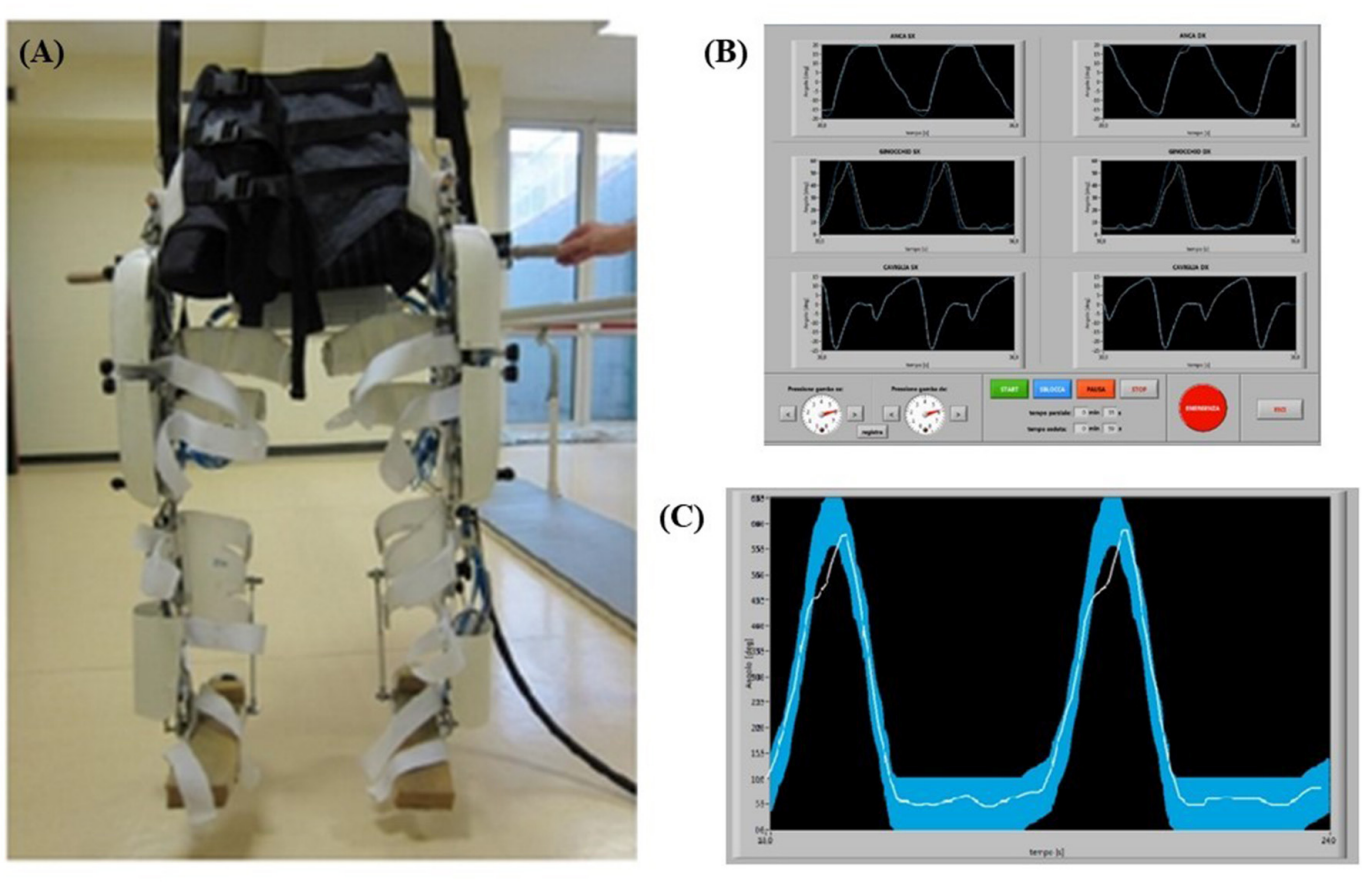
**(A)** P.I.G.R.O. orthosis used in the rehabilitation training. **(B)** P.I.G.R.O. operator's monitor screenshot with the six joints visualized. **(C)** Examples of biofeedback monitor. In particular, the thicker curve is the machine reference for the patient, while the thinner curve represents the patient's performance during the test.

The robotic orthosis used in this rehabilitation protocol is called P.I.G.R.O. and it was developed in collaboration between the Department of Psychology and the Department of Mechanical and Aerospace Engineering of the Polytechnic University of Turin. PIGRO consists of a modular and size-adaptable 6-DoF exoskeleton that can be adapted to patients with an anthropometric range between 10%ile woman and 95%ile man and is designed to reproduce the physiological movements of walking. It consists of two independent “legs” composed of three parts: Hip joint, femoral segment and tibial segment, which are adjustable in length according to the patient's measurements and involves six joints (two for the hip, knee and ankle) that can be monitored (Figure 1). Part of the orthosis structure uses spring steel so it is more comfortable and wearable (Belforte et al., 2014).

It can be used by patients either being completely passive, which is typically the case at the beginning of rehabilitation training, and then gradually active, which means that patients must gradually exert more effort and strength to perform the movement. The orthosis was used in conjunction with a Body Weight Support (BWS) system, allowing for complete weight loss of the mass of the orthosis itself and of the patients' body. The BWS provided during training was initially set to fully support the subjects' weight and gradually adjusted based on the individual's abilities. Therefore, rehabilitation training could be performed in complete suspension to avoid spasticity of the lower limbs, allowing the passive leg movement that induce a proprioceptive and kinesthetic activation. To ensure that the movement of the machine was transmitted and perceived correctly by the patient, it was necessary for the exoskeleton and the subject to adhere well to each other. This was guaranteed by the presence of orthopedic splints and Velcro straps along each joint and by a fabric corset on the abdominal area. The corset was divided into two independent sections, each one connected to a leg. If necessary, pads could be added to the parts in contact with the patient to improve comfort during use in suspension (Figure 1A).

The operator has at his disposal a computer with two monitors to control the machine: from the screen of the main one he can set the speed of the step cycle and adjust the pressure independently in the two limbs (to allow passive or active movement of the patient), it can start, pause and stop the movement, as well as being able to enter all the information related to the patient and the session in progress; moreover, in this window it is possible to observe the motion curves set in the machine for each articulation (Figure 1B). Specifically, two motion curves were observed during the participant movement for each articulation: one represents the movement run by the machine and the other represents the movement performed by the patient (Figure 1C). Those two curves were superimposed to allow an online comparison and a possible correction by the operator. In addition, participants were shown the motion curve of one joint of one or both legs at a time on a monitor to make them constantly aware of their motion performance through visual biofeedback and to allow them to better associate the correct movement with the proprioceptive information.

### 2.3. Experimental protocol

The experimental protocol proposed consisted of two types of training: physical, which combined classic physiotherapeutic rehabilitation and rehabilitation with robotic devices; and cognitive, which included rehabilitation through motor imagination and focus of attention.

**Figure 2:**
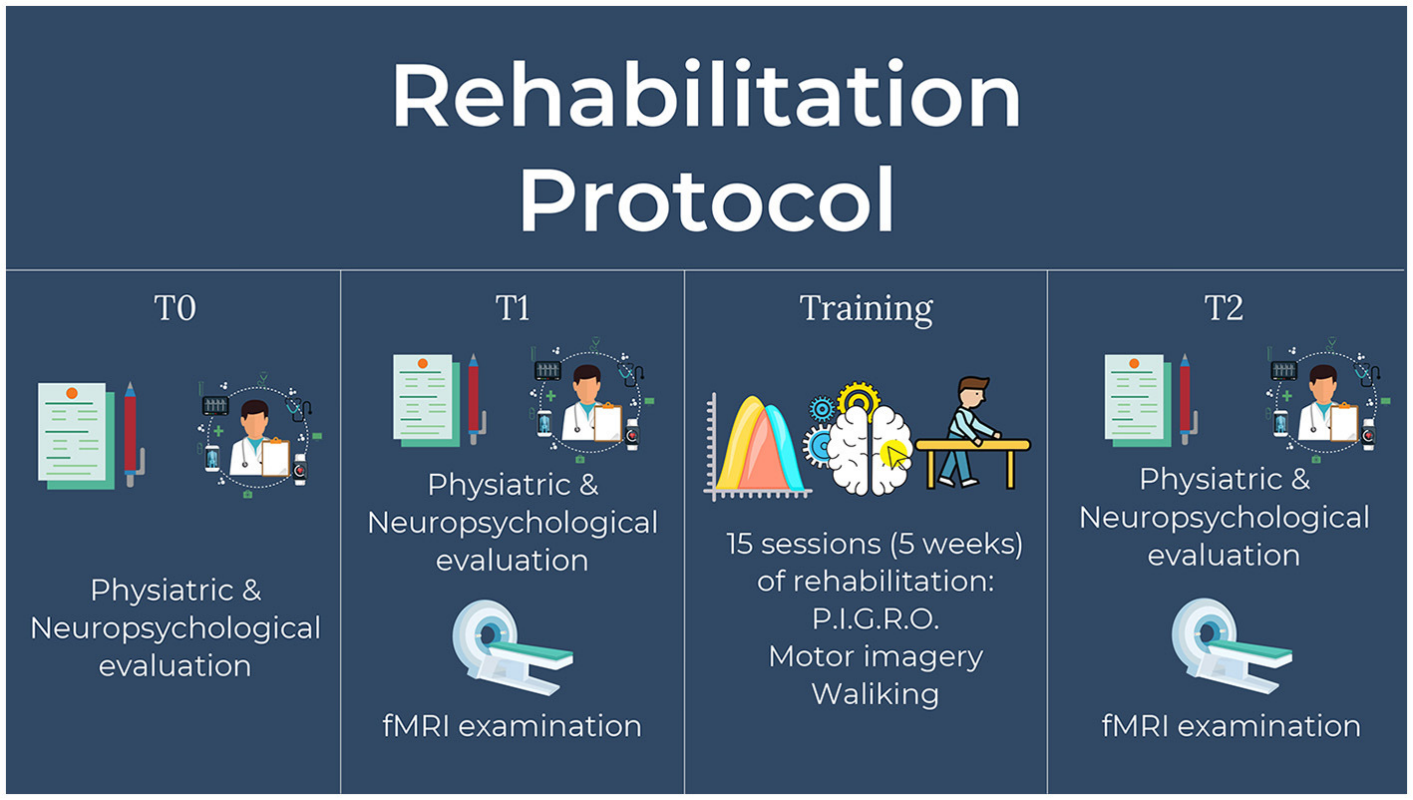
Schematic description of experimental design divided in the three time-points.

Patients underwent three sessions of physical and cognitive assessment. A first evaluation was made 5 weeks before the start of treatment (T0), one at the beginning of the treatment (T1), and one at the end of the treatment (T2). Concerning the physical evaluation the following tests were performed: Berg Balance Scale (BBS) (Berg et al., 1992) and Tinetti Balance and Gait Scale (TBGS) (Tinetti et al., 1986). Moreover, a neuropsychological evaluation was performed in order to check for the exclusion criteria and assess the preserved imagery ability. In T1 and T2 compatible subjects underwent a functional magnetic resonance examination (Figure 2).

#### 2.3.1. Physiatric evaluation

We used two of the most common scales (i.e., BBS and TBGS) that can provide an objective measure of walking ability from a behavioral perspective. Both scales were performed by a physical therapist at each of the experimental time points (T0, T1, and T2). BBS is used to assess balance and fall risk in both elderly healthy subjects and patients with various diseases (stroke, Alzheimer's disease, dementia, multiple sclerosis, Parkinson's disease). It consists of 14 items that examine the patient's ability to hold a position, move from one position to another, and complete a task. BBS scores are categorized according to the performance of functional status and independence into three levels: (a) 0 to 20, available mobility although wheelchair bound; (b) 21 to 40, walking with assistance; and (c) 41 to 56, walking independently (Berg et al., 1992). The TBGS assesses balance and gait, key factors in predicting fall risk, by evaluating motor skills that are important for daily living, such as standing up and sitting down. It consists of two different scales: the first part, which evaluates balance functions, consists of 9 tests that examine static balance abilities in different positions and the execution of stance changes. The second part, on the other hand, evaluates walking functions based on gait characteristics such as symmetry, step length, and continuity, using 10 items.

#### 2.3.2. Neuropsychological evaluation

Neuropsychological assessment includes evaluation of various cognitive domains and has been used to screen exclusion criteria. It includes the Mini Mental State Examination (MMSE) (Folstein et al., 1975), a neuropsychological test used to assess intellectual performance and the presence of cognitive impairment; the Token Test (short form of the Achen Aphasia Test) (De Bleser et al., 1986), used to assess some aspects of oral language comprehension and check for aphasia; Albert's Test (Fullerton et al., 1986), which is used to determine the presence of unilateral spatial neglect and it is specific for stroke patients; Frontal Assessment Battery (FAB) (Hurtado-Pomares et al., 2018), which is used to examine global executive functions at cognitive and behavioral levels. Finally, Short Form 36 Health Survey Questionnaire (SF-36) is a self-administered test that was used to capture the impact of a disease on various dimensions of quality of life such as physical functioning, limitations due to physical health, limitations due to emotional problems, energy and fatigue, emotional wellbeing, social activities, pain, and perception of general health. Moreover, the preserved imagery ability was evaluated through the use of Kinesthetic and Visual Imagery Questionnaire (KVIQ) (Malouin et al., 2007), a motor imagination questionnaire adapted for patients who are unable to stand or perform complex movements, in which both visual and kinesthetic dimensions of motor imagination are tested, and the Vividness of Visual Imagery Questionnaire (VVIQ) (McKelvie, 1995), which explores the vividness of visual imagination.

#### 2.3.3. Rehabilitation protocol

**Figure 3:**
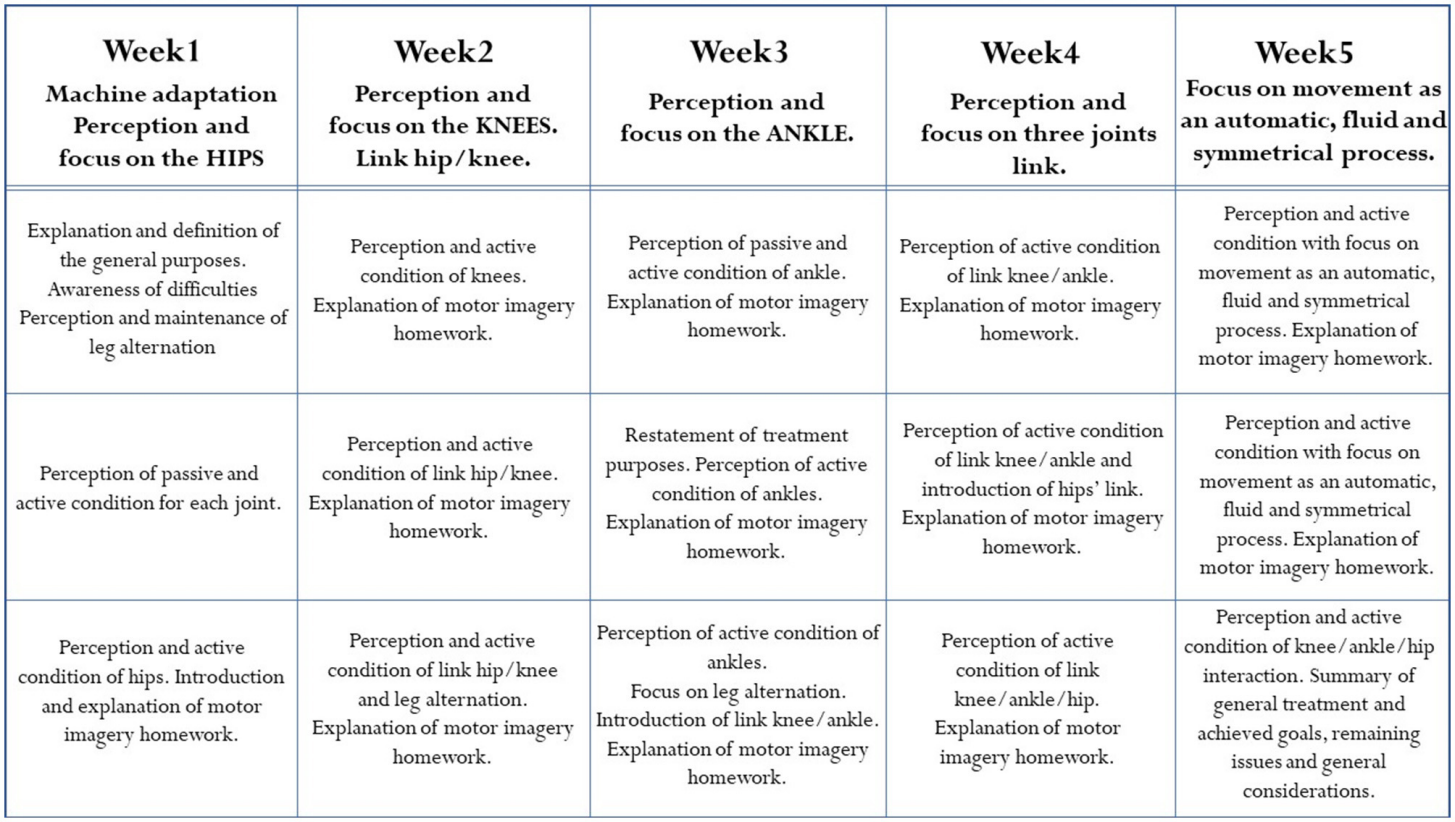
Aims and description of the rehabilitation protocol, organized in three sessions per week over a period of 5 weeks.

The rehabilitation protocol consisted of three sessions per week over a 5-week period. Each session lasted 1 hour and a half and was divided into three parts: The first took place on the robotic orthosis (30 min), in the second a motor imagery guided practice was performed, and in the last part the patients were asked to walk on the floor with the help of a physiotherapist. In addition, between sessions the subject was instructed to do motor imagery exercises at home, reinforcing the work done with the therapists. The detailed program of each session of the rehabilitation protocol are briefly described and summarized in Figure 3. The 15 sessions were divided into blocks: after getting used to the exoskeleton and the protocol in general, they focused on a specific joint (in the order hip-knee-ankle). The movement of each joint on the floor was presented and explained in its dedicated session. The movement curves in suspension were presented so that patients could perform them. When performing the movement, patients were asked to focus their attention first on the passive movement (i.e., when the pressure in the legs is high: 6 bar, the patient must not make efforts and must concentrate on the movement performed by the machine) and then on the active movement (i.e., when the pressure in the legs is low: 4 or 2 bar). In this active part, patients were asked to try to reproduce the movement, relying mainly on proprioception, but also on the verbal feedback from the psychologist, which becomes less and less as the rehabilitation progresses, and on visual feedback when needed. Even though the protocol consists of precise phases and tasks, sessions could vary according to the different needs of each patient. In particular, pressure could be changed from 4 to 2 bar, and a different amount of time, in a range between 1 and 3 min, could be dedicated to a specific joint, depending on patients' capabilities. We opted for a compromise between a reproducible protocol assuring methodological rigor and the respect for individual clinical needs: although it can be considered as a limitation of the study, tailorizing protocols represents a current gold standard in medicine and rehabilitation.

### 2.4. fMRI procedure

Eligible subjects (N = 6) underwent two sessions of fMRI examination in T1 and T2. All the subjects have a left hemisphere lesion. The task adopted in this study was inspired by the task used by Dobkin et al. (2004) for rehabilitation of ankle joint movement, as ankle movement appears to activate very similar brain networks to those activated during gait (Sahyoun et al., 2004).

The task was performed using a block design with 12 s of rest alternating with 12 s of active condition. Throughout the session, an image of two feet was projected: In the resting phase, the feet were white and the patients had to remain still, while in the active phase, the right or the left foot turned red and they had to move the corresponding foot in the first run, or imagine moving it in second run, performing plantar flexion and then dorsiflexion, like they were pressing a pedal.

Data acquisition was performed at the Koelliker hospital in Turin on a 1.5 T Intera scanner (Philips Medical Systems). Structural and functional images were acquired for each patient. In the first part of the exam a set of three-dimensional high-resolution T1-weighted structural images was acquired (FFE sequence, TR = 25 ms, TE = shortest, flip angle = 30°, acquisition matrix = 288 × 288, FoV = 288 mm). The set consisted of 107 sagittal contiguous images (slice thickness = 1,5 mm) covering the whole brain with an in-plane resolution of 0.99 mm × 0.99 mm. Functional T2-weighted images were acquired using echoplanar (EPI) sequences (TR = 3,000 ms, TE = 60 ms, flip angle = 90°, acquisition matrix = 64 × 64; FoV = 256 mm. For each task, a total of 206 volumes were acquired. Each volume consisted of 25 axial slices (slice thickness = 4 mm with a 0.5-mm gap), parallel to the anterior–posterior (AC–PC) commissure line and covering the whole brain.

### 2.5. fMRI analysis

Analyses for fMRI data were performed using the AFNI (Cox, 1996) and FSL (Jenkinson et al., 2012) software programs. Structural images were brain extracted, corrected for intensity bias, and spatially normalized to the Montreal Neurological Institute (MNI) space with non-linear registration. All functional volumes were slice timing corrected, spatially realigned to the first volume of the functional acquisition, and correct for scan motion. All functional volumes are then spatially smoothed with a 6 mm full-width half-maximum isotropic Gaussian kernel (FWHM) and the signal was normalized (center: 0; variations in %). Spatial parameters are computed for aligning average EPIs to high resolution T1w and then re-sample in the size of the functional acquisition (4 × 4 × 4.5 mm^3^) using a weighted sinc-interpolation method.

The fMRI responses of each subject were modeled using the General Linear Model (GLM). The design matrix of the GLM included the onset and duration of each experimental condition (12s) as well as the six predictors obtained from the motion correction parameters in the realignment process to account for the voxel intensity variations caused by the subjects' head movements. The conditions predictors were modeled as consecutive and convolved with a double gamma hemodynamic Hemodynamic Response Function (HRF).

As regard the group level analysis, results were transformed into standard space, a common brain mask was created (including all the standardized masks for each subject) and a paired-sample *t-*test was performed in order to compare activity before and after the rehabilitation protocol (T0 vs. T2) for each foot.

## 3. Results

### 3.1. Behavioral results

**Figure 4:**
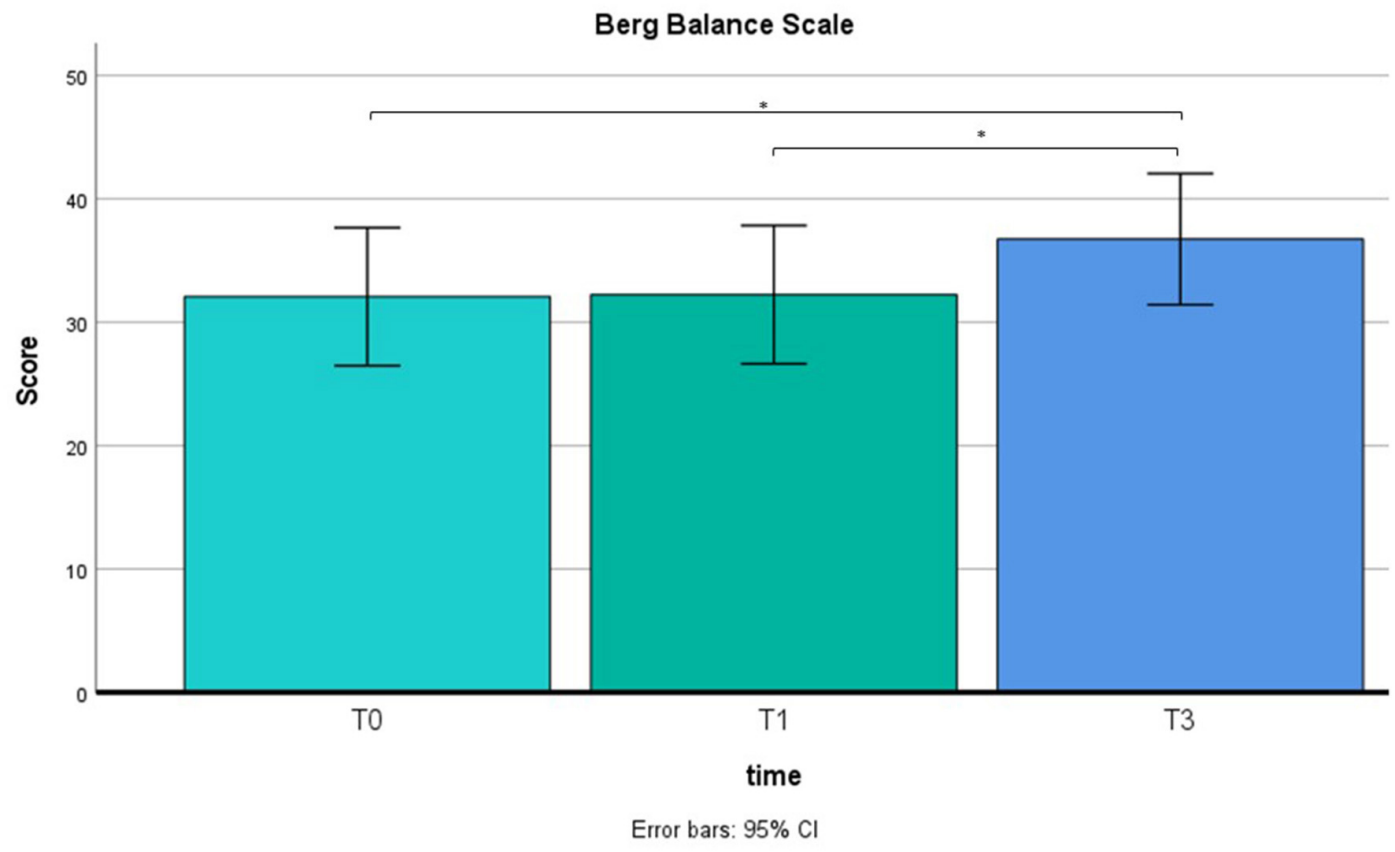
BBS results in T0 (mean = 32; SD = 8.5), T1 (mean = 32.2; SD = 9) and T2 (mean = 36.8; SD = 8.5). ^*^p < 0.05.

**Figure 5:**
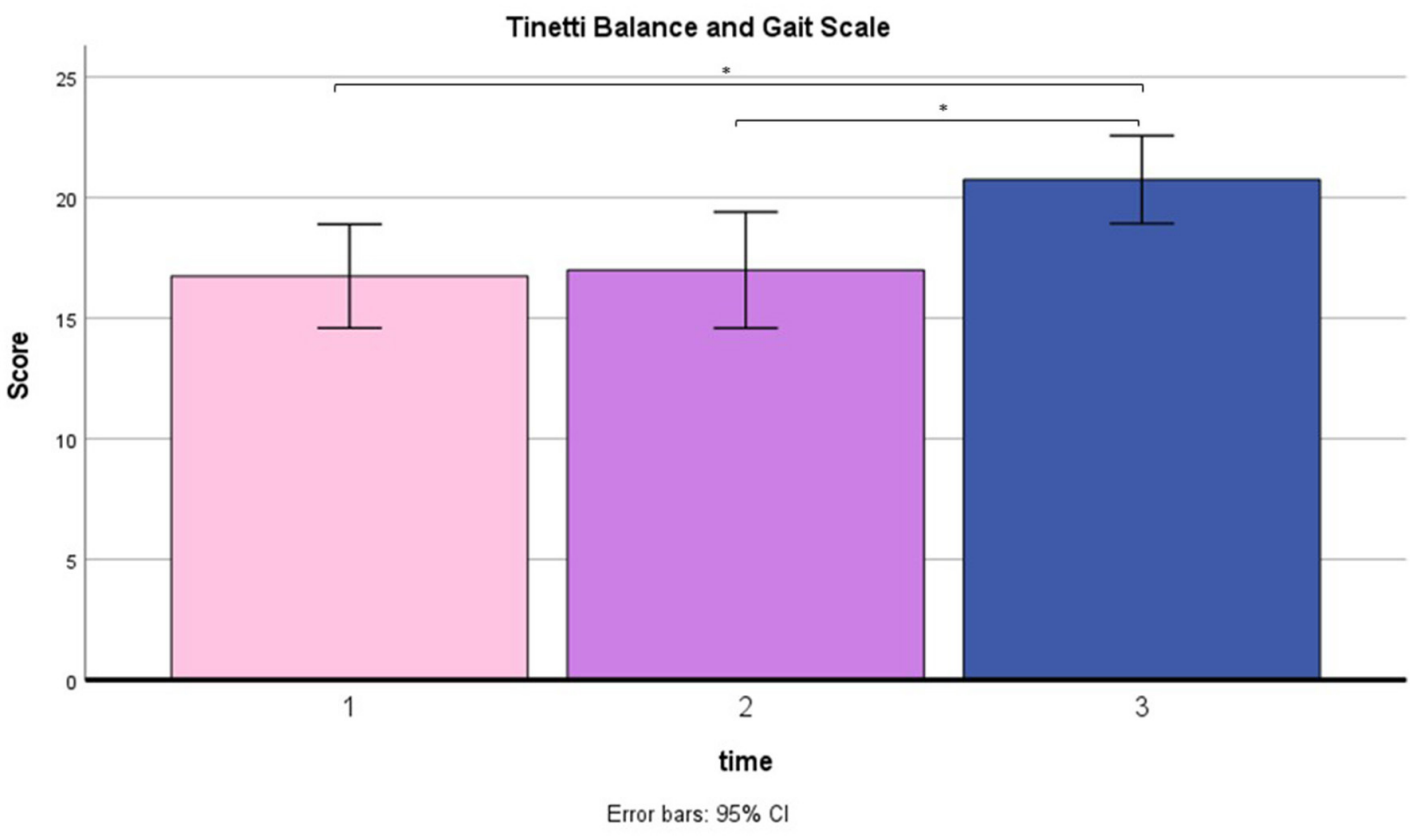
TBGS results in T0 (mean = 16.8; SD = 3.4), T1 (mean = 17; SD = 3.8) and T2 (mean = 20.8; SD = 2.8). ^*^p < 0.05.

A repeated measure ANOVA was used to compare pre-training with post-training on BBS and TBGS scores. The BBS was used to assess balance and stability of walking, and the TBGS was used to measure movements, postural changes and walking aspects related to a safe and efficient execution of the activities of daily living. ANOVA results showed a main effect on time (T0 vs. T1 vs. T2) in both tests (BBS: F = 7.5, *p* < 0.05; TBGS: F = 5.8, *p* < 0.05) so a paired-sample *t*-test was performed. Results are shown in Figures 4, 5. Results of *t*-test show on both scales that the group of patients (N = 12) presents an absence of changes between T0 (baseline) (BBS mean = 32.2 ± 8.5; TBGS mean = 16.8 ± 3.4) and T1 (BBS mean= 32.2 ± 9; TBGS mean = 17 ± 3.8, *p* > 0.05), confirming the stability of their conditions. On the contrary, a statistically significant improvement emerged between T1 (pre-training) and T2 (post-training) (BBS mean = 36.89 ± 8.5; TBGS mean = 20.8 ± 2.8) showing that our training positively affected their balance (*p* < 0.05).

### 3.2. fMRI results

Considering the size of fMRI sample (N = 6), the high variability of people's brain plasticity mechanism and of the lesions of participants in terms of their extent and location, we considered results with an uncorrected *p* value ≤ 0.05. This is a major limitation of the study. However, we still believe that the results obtained by fMRI analysis, represent a preliminary though promising finding, which may be an interesting starting point for future research.

#### 3.2.1. Motor activity

**Figure 6:**
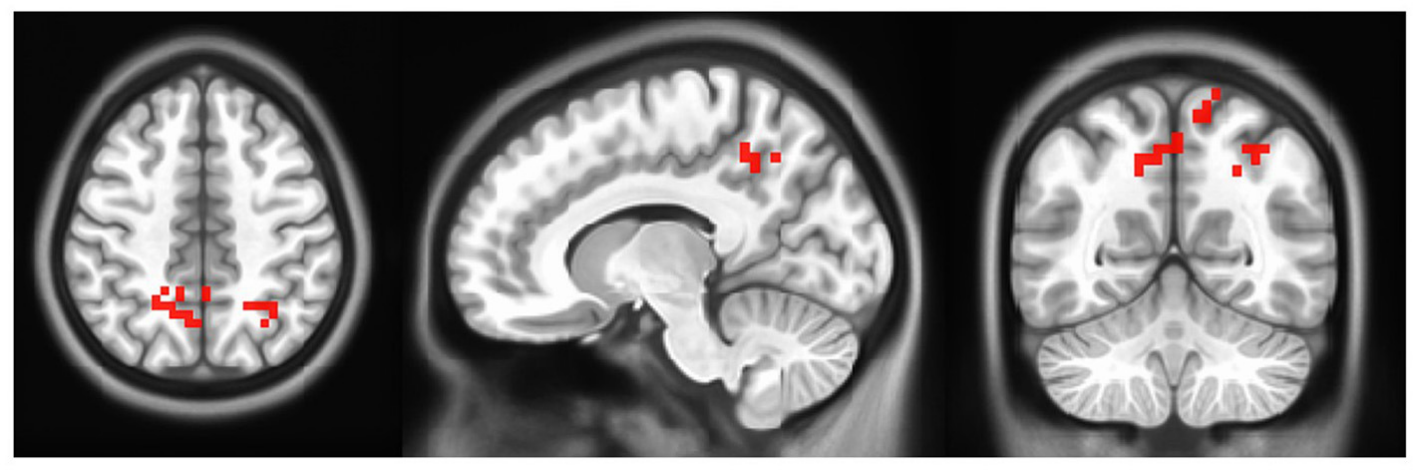
Group level activation in motor activity in post- minus pre- rehabilitation of right foot. Each activation is fully described in Table 2 (Left=Left).

A paired-samples *t-*test was performed to compare pretraining activity with post-training activity. The results of the paired-samples *t-*test on right foot motor activity comparing pre- and post-rehabilitation time at the group level (*N* = 6) show greater activation of activity after training compared to pre-training time in the bilateral precuneus, right angular gyrus (AG), and bilateral superior and inferior parietal lobule (SPL, IPL) (uncorrected *p* ≤ 0.05). The map of the threshold results is shown in Figure 6. Results are clustered for groups of voxels ≥10. In addition, the results are summarized in Table 2. No activation was found for the left foot using the same statistics.

**Table 2:** Differential motor activations between pre and post training in the right foot.

**Right foot**	**Anatomical area**	**%**	**Center of mass**			**Peak**		
	**Post minus Pre**		**X**	**Y**	**Z**	**X**	**Y**	**Z**
	L Precuneus	42.1%	4.7	55.9	50	-2	62	54
	R Precuneus	29.2%						
	L superior parietal lobule	6.5%						
	R superior parietal lobule	5.5%						
	L inferior parietal lobule	1.5%						
	R angular gyrus	48.3%	−31	60.1	46.8	−26	58	40.5
	R superior parietal lobule	19%						
	R inferior parietal lobule	4%						

Medicine Unit: Koelliker Hostpital, Turin. Uncorrected *p* ≤ 0.05; Cluster size ≥10. Intersection with CA_ML_18_MNI atlas.

#### 3.2.2. Imagery activity

The paired-sample *t-*test results on imagery activity comparing pre- vs. post- training at group level reveal a greater activation of those areas involved in motor execution and motor planning, especially when considering the right foot activity. In particular, precentral and postcentral gyrus, paracentral lobule, Supplementary Motor Area (SMA), and parietal lobules. Imagery results are summarized in Tables 3, 4.

**Table 3:** Differential imagery activations between pre and post training in the right foot.

**Right foot**	**Anatomical area**	**%**	**Center of mass**			**Peak**		
	**Post minus Pre**		X	Y	Z	X	Y	Z
	R SMA	43.9%	-11.1	-3.4	47.9	-6	10	49.5
	R middle Cingulate Cortex	27.6%						
	R Superior Frontal gyrus	14%						
	R SMA	26.3%	-1	23.1	63.2	14	30	63
	Bilateral Paracentral Lobule	39.2%						
	Bilateral Precentral gyrus	6%						
	Bilateral Postcentral gyrus	6%						
	L Superior Parietal Lobule	95.4%	21.5	65	49.3	22	62	45
	L Middle Occipital gyrus	3.6%						
	R Superior Parietal Lobule	55.7%	-15.5	56	61	-18	54	58.5
	R Precuneus	44.3%						
	**Pre minus Post**							
	L Thalamus	51.6%	12.6	29.7	11.8	14	26	9
	L Hippocampus	5.9%						
	L posterior Cingulate Cortex	2%						

Medicine Unit: Koelliker Hostpital, Turin. Uncorrected *p* < 0.05; Cluster size >10. Intersection with CA_ML_18_MNI atlas.

**Table 4:** Differential imagery activations between pre and post training in the left foot.

**Left foot**	**Anatomical area**	**%**	**Center of mass**			**Peak**		
	**Post minus Pre**		X	Y	Z	X	Y	Z
	R Insula Lobe	44.2%	−39.3	2	−10.4	−38	2	−9
	R Amygdala	6.9%						
	R Superior Temporal gyrus	3%						
	R Middle Frontal gyrus	47.0%	−22.6	−43.8	22.7	−18	−42	18
	R Superior frontal and medial gyrus	14.8%						
	Bilateral precentral gyrus	6%						
	R anterior cingulate cortex	10.8%						

Medicine Unit: Koelliker Hostpital, Turin. Uncorrected *p* < 0.05; Cluster size >10. Intersection with CA_ML_18_MNI atlas.

## 4. Discussion

In the present study, we tested a combined (physical & cognitive) protocol, directed to rehabilitate the gait of chronic stroke patients, using an original robotic exoskeleton, called P.I.G.R.O. The results suggest a significant overall improvement in gait performance associated with possible brain reorganization.

From a behavioral point of view, although we do not have certainty of durability over the long term, the BBS and TBGS scores showed an improvement in balance and walking ability after the rehabilitation. As indicated in Figures 4, 5, the group of 12 patients demonstrated stability in their conditions between the baseline (T0) and pre-treatment (T1) assessments on both scales. The BBS mean scores in T0 and T1 indicated that patients needed mobility assistance (Neuls et al., 2011). Although the mean score at T2 is still below the threshold of good balance (41/56) (Blum and Korner-Bitensky, 2008), indicating the inability to move safely, a significant improvement was observed between the pre-training (T1) and post-training (T2) assessments. These findings show that our training had a positive impact on patients' balance. Moreover, prior to training, the patients scored below the cutoff value (cutoff = 18) on the TBGS scale. Since this scale is highly sensitive to fall risk, values over the cutoff reflect the patients' high risk of falling, indicating insufficient efficiency and stability in their motor activities (Scura and Munakomi, 2023). After the training, their score exceeded the cutoff value, showing that they had recovered mobility to the point of significantly reducing the risk of falling. Altogether, these behavioral results indicate that the combined use of MI associated with a motor rehabilitation plan can be an effective protocol in inducing a motor performance improvement.

The imagination of a movement may be realized from two different perspectives: through an external/allocentric perspective (in the third person), or internally, through an egocentric perspective (in the first person) (Malouin et al., 2007). When individuals intentionally focus their attention on imaging to perform a movement in the first person, as realized in the present protocol, the mental representation of such movement is activated, supporting the reconstruction of its motor schema (Jeannerod, 1994; Sacco et al., 2011). The focus of attention has been suggested to be a crucial aspect in inducing neuroplasticity (Li et al., 2018). Moreover, the visual feedback plays an important role helping the construction of the proprioceptive experience (Ernst and Bülthoff, 2004). Although visual feedback in 3D might have been more effective (Song et al., 2015), the 2D online feedback here proposed helps to better associate movement with proprioceptive sensation by providing visual support in processing somatosensory feedback. Future studies could explore this further, for example, with the help of Virtual Reality devices, which has proven useful in many aspects of rehabilitation to make settings as ecologic as possible (Semblantes et al., 2018; Kim et al., 2020).

Importantly, our results are in line with previous studies, showing that the use of MI in motor rehabilitation protocols is effective in supporting the rehabilitation of motor deficits (Jackson et al., 2001; Dunsky et al., 2008; Zhang et al., 2011). It has been shown that stroke patients undergoing MI associated with motor-based therapy showed significant improvements in gross motor function of upper limbs (Machado et al., 2019). Moreover, a similar protocol that combined sensory-motor training and MI tested in a study with two cranial trauma patients, provided promising results in restoring balance and gait as well as enhancing connectivity in the motor network (Sacco et al., 2011). Although we cannot isolate the contribution of MI because we do not have a specific control group, we suspect, in agreement with previous studies, that this is one of the factors that contribute to the results since it is an integral and important part of the rehabilitation protocol.

In line with previous studies, the present training program seems to be able to improve patients' functional abilities, reducing their risk of falls. This may allow them to perform activities of daily living more safely and efficiently increasing independence in daily and social activities. Accordingly, the employment of a combined MI+P.I.G.R.O. protocol may have important implications for the quality of life and overall wellbeing of patients and their families. However, it cannot be speculated whether these improvements remain constant in the long term, i.e., beyond the 5 weeks considered in our study.

From a neurophysiological perspective, the improved gait performance appeared to be supported by a brain reorganization in the lesioned hemisphere during the motor task, particularly in higher-level motor networks. fMRI results show greater activation in the bilateral pre-cuneus, right AG, and bilateral SPL and IPL, when compared to before training activation for the contralateral side (i.e., right foot). Given the connectivity between the pre-cuneus and M1 (Zhang et al., 2011), its increased activation may reflect a general enhancement of the cerebral network involved in movement-related activities. More specifically, previous studies demonstrated the pre-cuneus involvement in higher-order aspects of action and its function as an interface between cognition and action, as described by Wenderoth et al. (2005). Similarly, AG acts as an integrative hub for multisensory information due to its rich connectivity. Previous studies have reported the role of AG during the learning process (Seghier, 2013), including motor skills learning (Draganski et al., 2004; Amad et al., 2017). More specifically, AG is one of the brain areas showing greater plasticity when learning challenging visuomotor tasks (such as, juggling), and, as observed in a resting state connectivity study, it was demonstrated to be included in a neural network deeply involved in motor training skills (Draganski et al., 2004; Amad et al., 2017). Moreover, activation in the bilateral SPL may reflect the enhanced kinesthetic process. SPL appears to be involved in another high-level mechanism, that compares the spatial position and orientation of the stored representation of the motor plan with afferent signals (Wolbers et al., 2003). Remarkably, no cerebral activation was observed during left foot movement in the post- minus pre- contrast, suggesting that cerebral reorganization occurred only in the lesioned hemisphere. Taken together, this evidence, when compared with the current literature, seems to indicate that the brain changes associated with motor activity occur in areas involved in higher-level aspects of motor control, that is, motor integration and association.

Importantly, looking at the MI activity results for the right foot, it seems that MI training effectively stimulates the motor networks that we expected to be involved in rehabilitation. The brain activity during the imagery task seems to be enhanced by the training, especially for the lesioned hemisphere. Although the involvement of primary motor cortex in MI has been considered controversial by the literature (Dechent et al., 2004), recent studies exploiting transcranial magnetic stimulation indicated that MI is able to improve primary motor cortex plasticity in healthy subjects (Avanzino et al., 2015). Apparently, motor imagery leads to the activation of the same brain areas as actual movement both in healthy subjects and in neurological patients (Hallett et al., 1994; Gerardin et al., 2000; Hanakawa et al., 2003; Kimberley et al., 2006; Confalonieri et al., 2012). Importantly our results may be considered as supporting evidence to this hypothesis. In particular, we found activation of primary motor and sensory cortices, paracentral lobule, which controls motor and sensory innervation of the contralateral lower extremity, SMA, involved in planning motor sequence and movement execution, and parietal lobules, responsible for transforming motor information into motor commands. As suggested by previous works, a possible interpretation is that imagery training promotes the kinesthetic sensation of movement and allows better activation of processes involved in the motor task, serving as a primer that stimulates the actual motor network (Jackson et al., 2001). A recent TMS work provides further proofs toward the role of imagery in motor learning and plasticity assuming its actual occurrence at cortical level (Ruffino et al., 2019). In conclusion, although we do not have a control group to verify the actual specific contribution of MI, we suggest that imagery training integrated into the rehabilitation protocol could be one of the factors that affected motor rehabilitation in our sample.

Overall, our preliminary findings suggest that the described rehabilitation protocol, matching the employment of P.I.G.R.O. exoskeleton with the use of MI, it can be effective in rehabilitating gait performance in chronic stroke patients and in supporting changes in the cerebral network involved in movement. However, given the sample size of the present research, further studies are needed to confirm the results. Ultimately, this research has the potential to support the development of new multidisciplinary approaches for stroke patients, improving their quality of life, and reducing the burden of stroke-related disability. Future research should further investigate the role of lesion extent and location in the recovery and the use of motor imagery in the context of stroke patient rehabilitation.

## Data availability statement

The raw data supporting the conclusions of this article will be made available by the authors, without undue reservation.

## Ethics statement

The studies involving human participants were reviewed and approved by AOU Città della Salute e della Scienza di Torino. The patients/participants provided their written informed consent to participate in this study.

## Author contributions

KS and GG conceptualized the work and the experimental design. KS, MZ, and SD contributed to patients' screening protocol and data acquisition. M-CV, KS, IR, and AC contributed to data analysis. M-CV, IR, and KS wrote the first draft of the manuscript. All authors contributed to manuscript revision, read, and approved the submitted version.
